# Emerging actionable targets to treat therapy-resistant colorectal cancers

**DOI:** 10.20517/cdr.2021.96

**Published:** 2022-01-04

**Authors:** Emanuela Grassilli, Maria Grazia Cerrito

**Affiliations:** Department of Medicine and Surgery, University of Milano-Bicocca, Monza 20900, Italy.

**Keywords:** Colorectal cancer, resistance, chemotherapy, target therapy, EGFR, ERBB2, MET, BRAF, kinase inhibitors, immune checkpoint inhibitors, gut microbiota

## Abstract

In the last two decades major improvements have been reached in the early diagnosis of colorectal cancer (CRC) and, besides chemotherapy, an ampler choice of therapeutic approaches is now available, including targeted and immunotherapy. Despite that, CRC remains a “big killer” mainly due to the development of resistance to therapies, especially when the disease is diagnosed after it is already metastatic. At the same time, our knowledge of the mechanisms underlying resistance has been rapidly expanding which allows the development of novel therapeutic options in order to overcome it. As far as resistance to chemotherapy is concerned, several contributors have been identified such as: intake/efflux systems upregulation; alterations in the DNA damage response, due to defect in the DNA checkpoint and repair systems; dysregulation of the expression of apoptotic/anti-apoptotic members of the BCL2 family; overexpression of oncogenic kinases; the presence of cancer stem cells; and the composition of the tumoral microenvironment and that of the gut microbiota. Interestingly, several mechanisms are also involved in the resistance to targeted and/or immunotherapy. For example, overexpression and/or hyperactivation and/or amplification of oncogenic kinases can sustain resistance to targeted therapy whereas the composition of the gut microbiota, as well as that of the tumoral niche, and defects in DNA repair systems are crucial for determining the response to immunotherapy. In this review we will make an overview of the main resistance mechanisms identified so far and of the new therapeutic approaches to overcome it.

## INTRODUCTION

Notwithstanding widespread, effective measures of preventive screening, early detection and major advances in treatment options, colorectal cancer (CRC) is still the fourth cause of cancer-related death worldwide^[1,2]^. In fact, due to the relatively asymptomatic progression of the disease in the early stages, patients are frequently diagnosed with metastatic CRC (mCRC), with a 5-year survival rate of around 14%^[3]^. Neo-adjuvant or adjuvant chemotherapy, together with surgery, represent the backbone of the therapeutic approach for both early-stage and metastatic cancer patients. Depending on tumor site and progression of the disease, chemotherapy can be associated with radiotherapy^[4-6]^ and, in selected cases, it can be possibly integrated with targeted therapy and/or immunotherapy. However, despite a variety of therapeutic approaches, resistance - both intrinsic and acquired - to drug treatment(s) remains one of the greatest challenges in the long-term management of incurable mCRC and eventually contributes to death^[7,8]^. In the last two decades, remarkable progresses have been made in the understanding of the mechanisms underlying drug resistance and in the unraveling of the feedback mechanisms fueling acquired resistance to targeted and/or immunotherapy. In addition, several phenotypes and synthetic lethality screens uncovered important liabilities that can be pharmacologically targeted in resistant tumors and in specific mutational settings^[9]^. Accordingly, hundreds of clinical trials are currently undergoing to test new drugs and new combinations for the treatment of mCRC. This review will pinpoint the most promising targets emerged in the last decade.

## RESISTANCE TO CHEMOTHERAPY

**Figure 1:**
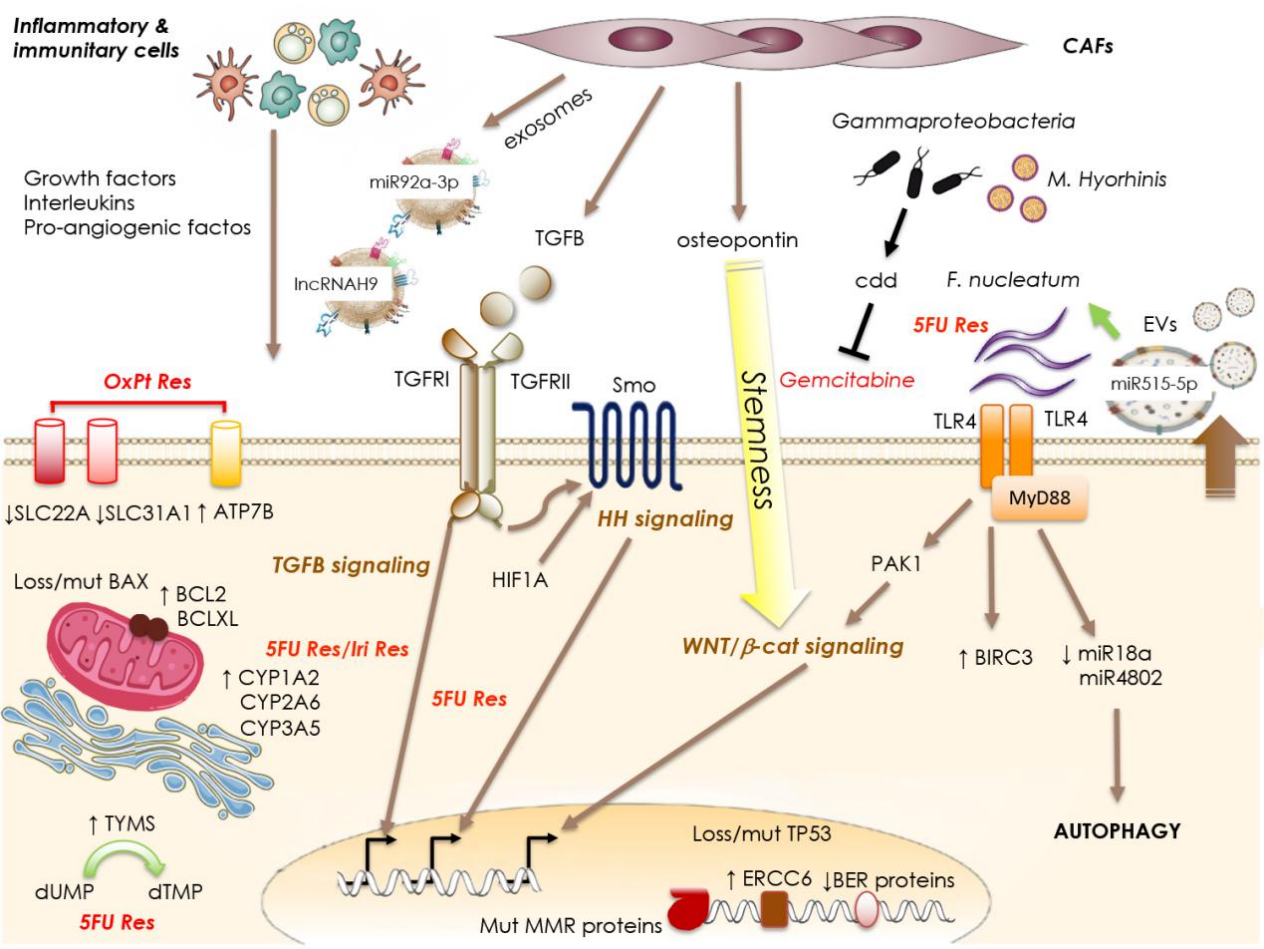
Mechanisms of resistance to chemotherapy. Tumor cell-intrinsic mechanisms may involve: dysregulated expression of drug transporters (SLC family members or ATP7B) and enzymes involved in drug metabolism (CYP family members), imbalanced expression of anti-/pro-apoptotic molecules (BAX mutation/loss or increased BCL2 expression), or dysregulation of DNA repair mechanisms and checkpoints (*TP53* mutation/loss, MMR proteins mutation, diminished expression of BER proteins, or increased ERCC6 expression). External signals acting on the tumor cells to trigger resistance may derive from the cells populating the tumoral niche such as the CAFs releasing TGFB, osteopontin and exosomes containing specific lncRNAs and miRs. In addition, inflammatory and immunitary cells of the niche release a number of interleukins, growth factors, pro-angiogenic factors. Also, specific components of the microbiota can contribute to the resistance to chemotherapy by directly inactivating the drug (Gammaproteobacterial or *M. hyorhinis*) or by engaging the TLR4/MyD88 system to transduce pro-survival and autophagic signals. SLC: Solute carrier; MMR: mismatch-repair system; CAFs: cancer-associated fibroblast.

According to the American Society of Clinical Oncology (ASCO) guidelines (https://www.asco.org/), the main drugs used for chemotherapy are 5-fluorouracil (5-FU), irinotecan, and oxaliplatin, either alone or in combinations such as in the FOLFOX (5-FU + leucovorin + oxaliplatin) or FOLFIRI (5-FU + leucovorin + irinotecan) regimens. 5-FU-based chemotherapy is the main approach for advanced CRC and, when used in combination, initial responses are up to 55%-65%. When administered sequentially, median overall survival (OS) can range from 18 to 20 months^[10]^. Although 5-FU initially de-bulks the tumor mass, recurrence usually occurs, thus pinpointing how acquired drug resistance, and consequent tumor re-growth, represents the main obstacle to effective clinical outcomes for CRC patients. Several different mechanisms have been identified accounting for resistance to 5-FU-based chemotherapy^[11]^ and increasing number of strategies are being explored to increase the responsiveness of resistant tumors to chemotherapy [Figure 1].

### Dysregulated expression of drug transporters and enzymes involved in drug metabolism

Plasma membrane transporters are pivotal for the uptake into and the efflux out of the cells of endogenous and exogenous molecules. They are divided in two big families: the ATP-binding cassette (ABC) family and the solute carrier (SLC) family. Members of both families serve a range of physiological roles but some of them also are determinants of drug disposition via affecting absorption, distribution, and excretion of drugs [Table 1].

**Table 1:** Dysregulated expression of drug transporters and enzymes involved in drug metabolism

**Gene**	**Protein function**	**Correlation with drug resistance**	**Ref.**
*SLC22A3*	Uptake of platinum compounds	Lower SLC22A3 expression correlates with worst PSF in patients receiving FOLFOX6 regimen	[20]
		Post-treatment intracellular concentration of OxPt is higher in SLC22A3-overexpressing cells; upregulation of SLC22A3 in mouse xenografts rendered tumors more responsive to OxPt treatment	[21]
*SLC31A1*	Copper influx transporter, involved also in OxPt intake	SLC31A1 level predicts prolonged survival and enhanced response to platinum-based regimens in cancer patients with several epithelial cancers	[24]
*ATP7B*	Copper efflux transporter, involved also in OxPt efflux	Increased levels of ATP7B are associated with poor outcome in CRC patients receiving oxaliplatin-based chemotherapy	[23]
*CYP1A2*/*CYP2A6* *CYP3A5*	Cytochrome P450 enzymes involved in drug metabolism	Increased expression in 5-FU-resistant HCT116 CRC cell line; addition of the CYP450 inhibitor phenylpyrrole enhanced 5-FU-induced cytotoxicity in 5-FU-resistant cells	[25]
		Higher intratumoral expression of CYP3A5 in patients with CRC who do not respond to irinotecan-based chemotherapy	[29]
*TYMS*	Enzyme that maintains the dTMP pool critical for DNA replication and repair and is inhibited by 5-FU	Increased expression of TYMS in pretreatment tumor biopsies identified tumors non-responsive to 5-FU-based therapy	[26]

PSF: Progression-free survival; OxPt: oxaliplatin; 5-FU: 5-fluorouracil; FOLFOX: 5-FU + leucovorin + oxaliplatin.

Since the mid-1980s several members of the ABC family have been deemed to be play an important role in the resistance to chemotherapy, given that their dysregulated expression may lead to a decreased uptake or an increased efflux of anticancer agents^[12-14]^. Even though roughly half of the members have been shown to efflux anticancer agents in some context, the use of cell lines with high ABC transporter expression levels might have led to an overestimation of their role in cancer^[15]^. In addition, transporters are usually parts of more complex networks usually under the same transcriptional regulation; therefore, their upregulation may be more of an epi-phenomenon than of a cause-related effect^[16]^. A paradigmatic example of this are the data reported by Gao *et al*.^[17]^. They first showed that, in colon cancer cell lines made resistant to 5-FU, drug treatment induced the expression of different ABC transporters as well as the activation of IRE1, an enzyme involved in the unfolded protein response ensuing from endoplasmic reticulum stress^[18]^. Disabling the enzyme, by both a specific inhibitor and by RNA silencing, they observed a decrease in the expression of the transporters as well as the sensitization to 5-FU. Given that the unfolded protein response is a complex response where several genes are regulated downstream of IRE1^[19]^, their experiments do not rule out that the sensitization might be due to the suppression (or activation) of other components of the response. Therefore, the decrease in the expression of the transporters simply correlates with the sensitization effect without a clear cause-effect relationship having been established.

For decades now plenty of experimental data have been produced - both in *in vitro* and *in vivo* systems - showing that the increased expression of several members correlate with increased resistance to chemotherapeutic agents as well as their inhibition by specific inhibitors could restore drug sensitivity^[12,13]^. Although the substrates and key roles for most of these transporters have been identified, the extent to which these transporters play an effective role in clinical multidrug resistance has not yet been clarified^[15,16]^, not in general nor in the specific case of CRC. In fact, *ex-vivo* data are still pretty inconclusive: for example ABCB1 expression is generally low in tumors, but for few exceptions, and specific inhibitors have clinically failed^[16]^.

On the other hand, some members of the SLC family 22 shown to be involved in the uptake of platinum compounds have been more directly linked to drug efficacy. For example, in a retrospective study Gu *et al*.^[20]^ found that high expression of SLC22A3 (OCT3) may be a protective factor for CRC patients postoperatively treated with FOLFOX6 as a first-line adjuvant chemotherapy. In line with this, the same group also demonstrated in *in vitro* and *in vivo* experimental systems that the cellular concentration of oxaliplatin and its cytotoxicity were significantly increased in response to high expression of OCT3, whereas OCT3 knockdown directly increased the invasion and migration of colon cancer cells. In addition, upregulation of OCT3 expression in colon cancer xenografts via treatment with the DNA methyltransferase inhibitor decitabine increased the cellular concentration of the drug and improved its curative effect^[21]^. Key mediators for oxaliplatin accumulation inside the cells are also a series of proteins initially identified as copper transporters. It has been shown that the major copper influx transporter SLC31A1 (CTR1) regulates tumor cell uptake whereas the two copper efflux transporters ATP7A and ATP7B regulate the efflux^[22]^. Accordingly, it has been reported that increased levels of ATP7B are associated with poor outcome in CRC patients receiving oxaliplatin-based chemotherapy^[23]^, whereas a large meta-analysis of literatures and datasets performed by Sun *et al*.^[24] ^revealed that high CTR1 level predicts prolonged survival and enhanced response to platinum-based regimens in cancer patients with a number of epithelial cancers.

A change in the expression or activation of enzymes involved in drug metabolism by cancer cells can enhance the catabolism of the drugs or result in diminished activation of prodrugs. In both cases, the result is an impairment of the pharmacological action of the chemotherapeutic agent [Table 1]. For example, Untereiner *et al*.^[25]^ produced a 5-FU-resistant HCT116 CRC cell line characterized by a significant increase in the expression of the drug-metabolizing cytochrome P450 enzymes CYP1A2 and CYP2A6. Accordingly, they reported that the addition of the CYP450 inhibitor phenylpyrrole enhanced 5-FU-induced cytotoxicity in 5-FU-resistant cells^[25]^. Also, increased expression of thymidylate synthase (the enzyme inhibited by 5-FU) in pretreatment tumor biopsies could identify tumors that would be non-responsive to 5-FU-based therapy^[26]^. At variance, downregulation of thymidine phosphorylase (which plays an essential role in activating the oral prodrug capecitabine to 5-FU) causes resistance via insufficient drug activation^[27]^.

Irinotecan undergoes extensive metabolism in both the liver and the intestine; it is converted to inactive metabolites by CYP3A4 and CYP3A5^[28]^. Studying a CRC patient cohort, Buck *et al*.^[29]^ recently reported an association between the response to irinotecan and the expression CYP3A5; in fact, they found a significantly higher intratumoral expression of CYP3A5 in patients with CRC who do not respond to irinotecan-based chemotherapy and thus suggested a causal role of CYP3A5 in tumor resistance.

### Dysregulation of DNA repair mechanisms and checkpoints

5-FU and all the most used drugs for treating CRC act by inducing, directly or indirectly, DNA damage and consequently activate cell’s DNA damage response. Depending on the entity of the damage and on the functionality of the DNA damage response, apoptosis can be the outcome. In fact, several DNA repair mechanisms can intervene to remove or repair drug-induced damage thus avoiding cytotoxicity, and their dysregulation can contribute to the drug-resistant phenotype [Table 2]. For example, ERCC6 - a member of the excision repair cross-complementation (ERCC) family of enzymes, involved in the nucleotide excision repair pathway (NER) - is upregulated in CRC tissues compared to matched non-tumoral adjacent tissues and is also upregulated in patients resistant to 5-FU treatment. Conversely, low ERCC6 expression is associated with better response to chemotherapy and survival in CRC^[30]^.

**Table 2:** Dysregulation of DNA repair mechanisms and checkpoints

**Gene/system**	**Protein function**	**Correlation with drug resistance**	**Ref.**
***ERCC6***	Member of ERCC family of enzymes involved in the NER	Upregulated in CRC tissues compared to matched non-tumoral adjacent tissues Higher levels in patients resistant to 5-FU treatment low expression associated with better response to chemotherapy and survival	[30]
***BER***	Members of the BER system repairs bulky helix-distorting lesions	CRC patients with defects in components of the mismatch-repair system and expressing high levels of BER proteins have more aggressive tumors and poor outcomes after chemotherapy	[31]
***MMR***	Members of MMR are involved in recognizing and repairing erroneous insertion, deletion, and mis-incorporation of bases	dMMR patients with stages II and III CRC are less responsive, if not at all, to 5-FU-based chemotherapy whereas a better response is achievable in pMMR patients	[33,34]
***TP53***	DNA damage checkpoint	Loss or impairment of TP53 affects the response to chemotherapy in several *in vitro* and *in vivo* systems	[37]
		Loss or impairment of TP53 predict the poor response of patients with mCRC treated with chemotherapy	[38]
		Loss or impairment of TP53 associated with poor survival after FOLFOX therapyLoss or impairment of TP53 associated with poor survival after FOLFOX therapy	[39,40]

ERCC: Excision repair cross-complementation; NER: nucleotide excision repair pathway; BER: base-excision repair; MMR: mismatch-repair system; dMMR: defective MMR; pMMR: proficient MMR.

Activity of the base-excision repair (BER) system is pivotal in determining 5-FU-induced cytotoxicity in cells concomitantly defective for components of the mismatch-repair system (dMMR), as shown by the fact that CRC patients expressing high levels of BER proteins have more aggressive tumors and poor outcomes after chemotherapy^[31]^. Of note, zelpolib, a specific inhibitor of DNA polymerase δ an essential component of both NER and BER pathways - has been recently synthesized and shown to render cells sensitive to PARP inhibitors^[32]^. Therefore, it would be of interest to assess whether PARP inhibitors are cytotoxic in ERCC6-overexpressing and MMR/BER defective 5-FU-resistant models.

MMR status is particularly important in CRC given that around 15% of patients have one or more components (*MLH1*,* MLH3*,* MSH2*, or* MSH3*) mutated or silenced, often with a microsatellite instability (MSI) phenotype. Notably, patients with stages II and III CRC are less responsive, if not at all, to 5-FU-based chemotherapy whereas a better response is achievable in patients with MMR-proficient (pMMR) tumors^[33,34]^. In addition, MMR phenotype is also predictive of resistance to oxaliplatin-based chemotherapy^[35]^. Notably, collections of evidence are accumulating that dMMR and MSI-high (MSI-H) heavily pre-treated patients (at least two prior lines of therapy for metastatic disease) show a durable clinical benefit when treated with programmed cell death protein 1 (PD-1) inhibitors, particularly in the metastatic setting^[36]^.

The response to DNA damage is tightly controlled by DNA damage checkpoints and their misfunctioning may allow the survival of damaged cells and the selection of new mutations. In this case, the resistance to chemotherapy as well as the tumor progression are favored characteristics. The best-known DNA damage checkpoint, mutated or lost in 50% to 70% of CRC cases, is *TP53* which makes it the gene with the highest mutation rate in CRC^[37,38]^. Loss or impairment of *TP53* have been shown to affect the response to chemotherapy in several *in vitro* and *in vivo* systems^[37]^, to predict the poor response of patients with mCRC treated with chemotherapy^[38]^, and be associated with poor survival after FOLFOX therapy^[39,40]^.

Many promising synthetic lethal vulnerabilities, whose targeting kills p53-null drug resistant-resistant CRC cells, have been identified by an shRNA screen performed in our lab^[41]^. Several of them have successively been validated by other labs such as PIM-1^[42]^, TRIB3^[43]^, EPHA2^[44,45]^, CHK1^[46]^, and VEGFR2^[47]^. We focused our studies particularly on two targets: GSK3B and p65BTK. We showed that GSK3B is significantly more activated in drug-resistant *vs*. responsive CRC patients and is associated with cancer progression, poor response to therapy, and worse OS. Experimentally, we demonstrated that in the absence of p53, GSK3B activity allows cells to survive, despite treatment with DNA-damaging drugs, by sustaining DNA repair and that its downregulation restored sensitivity to 5-FU of p53-null colon cancer cells - both in *in vitro* and in *in vivo* models - via induction of regulated necrosis^[48]^. Moreover, we also demonstrated that GSK3A is redundant with GSK3B modulating drug resistance and chemotherapy-induced regulated necrosis^[49]^. A general role for GSK3B in sustaining resistance to chemotherapy has been validated also in other types of cancers and 9-ING-41, a novel GSK3B inhibitor developed by actuate therapeutics, is currently undergoing phase 1 and phase 2 trials, as a single agent and in combination with cytotoxic agents, in patients with refractory cancers, including CRCs^[9]^. p65BTK is a novel isoform of the Bruton’s tyrosine kinase we isolated from the screening and subsequently characterized. Compared to the known Bruton’s tyrosine kinase (BTK) expressed in bone marrow-derived cell lineages, p65BTK lacks a stretch of 86 amino acids at the N-term and is translated from an mRNA containing a different first exon. Protein abundance is regulated at the post-transcriptional level and under the control of RAS/ERK pathway. Moreover, p65BTK is endowed with strong transforming activity that depends on ERK1/2 and its inhibition abolishes RAS transforming activity. Accordingly, p65BTK overexpression in CRC tissues correlates with ERK1/2 activation^[50]^, histotype, and cancer progression^[51]^. p65BTK silencing or chemical inhibition affects the growth of CRC cells and overcomes the 5-FU resistance of p53-null CRC cells by abolishing a 5-FU-elicited TGFB1 protective response and triggering E2F-dependent apoptosis. In addition, the combination of BTK inhibitors with 5-FU is cytotoxic for p53-null patient-derived organoids and significantly reduced the growth of xenografted tumors, thus giving the proof-of-concept for suggesting the use of BTK inhibitors in combination with 5-FU as a novel therapeutic approach in CRC patients^[51]^. Further studies from our lab indicate p65BTK as an actionable target in different solid tumors other than CRC. In fact, preclinical data indicate that depending on the tumor type, BTK inhibitors used alone can induce cytotoxicity in gliomas^[52]^, be more effective than standard-of-care (SOC) chemotherapy in drug-resistant ovarian cancer^[53]^ or can kill drug-resistant tumor cells when used in combination with SOC chemotherapy or targeted therapy in non-small-cell lung cancer^[54]^, and melanomas (unpublished data).

Interestingly, its targeting is effective in cells with p53 loss and defects along the RAS/MAPK pathway, making it an actionable target for a broad range of solid tumors resistant to SOC chemotherapy. Notably several drugs targeting BTK are already in clinic for certain types of B-cell malignancies (e.g., ibrutinib, acalabrutinib, and zanubrutinib), whereas several others are in clinical trial for B-cell malignancies (e.g., orelabrutinib, spebrutinib, pirtobrutinib, and BGB-3111) and for different type of diseases characterized by excessive B-cell activation (e.g., remibrutinib, renebrutinib, tolebrutinib, and rilzabrunib). Thus, assessing their efficacy in drug-resistant CRCs and other solid tumors would be feasible and advantageous.

### Imbalanced expression of anti-/pro-apoptotic molecules

Drug resistance has been demonstrated to be associated with low levels of the pro-apoptotic BAX protein or its loss^[55,56]^, which can occur in CRC patients because of inactivating mutation or somatic frameshift mutation in the dMMR setting^[57]^. In a low-expressing experimental CRC model, its re-induction by using andrographolide, a natural diterpenoid, restored the apoptotic response to 5-FU of drug-resistant CRC cells^[58]^. Drug resistance has also been associated with increased levels of the anti-apoptotic BCL-XL protein, which largely occurs in CRC either by amplification or by overexpression^[59]^. Several molecules antagonizing BCL-XL, the so-called BH3-mimetics, are currently being tested in experimental models in combination with chemo- or targeted therapy. For example, ABT-737 has been shown to improve the response to oxaliplatin in a *TP53*wt/*KRAS*mut background^[60]^. Both ABT-737 and WEHI-539 have been shown to lower the apoptotic threshold by increasing the mitochondrial priming, thus sensitizing resistant CRC cells to chemotherapy; also, Ch282-5 potentiated the response to oxaliplatin both *in vitro* and *in vivo*^[61]^. On the other hand, ABT-263 has been proven to overcome hypoxia-driven radioresistance and improve radiotherapy^[62]^. Several BH-3 mimetics are currently being tested in clinical trials, even though not for CRC, but mainly for hematological malignancies and other solid tumors^[59]^. Hence, it may be hypothesized that in a near future these molecules will also be tested in CRC patients.

### Cancer stem cells and the tumoral niche

It has been repeatedly demonstrated that in the bulk of the tumor population, a very small fraction is represented by cancer stem cells (CSCs) that are intrinsically resistant to chemotherapy. In addition, upon exposure to anticancer agents and radiotherapy they may enter a quiescent state, resistant to anti-proliferative drugs; once the therapy is suspended, they can self-renew and differentiate into heterogeneous lineages of cancer cells, thus driving tumor recurrence^[63]^. Initially, several data indicated that CSCs upregulate drug-efflux pumps, have a superior DNA-repair capacity, and have enhanced antioxidant defenses^[64]^. More recently, cell plasticity and, in particular, the ability of CSCs to adopt a quiescent state have also emerged as important drivers of drug resistance. In fact, lineage-tracing approaches have revealed that the potential of committed cells to move up and down the hierarchy of differentiation (“plasticity”) is more widespread than previously thought. Interestingly, several studies have provided evidence that both CSCs and non-CSCs are plastic and capable of undergoing phenotypic transitions in response to appropriate stimuli^[64]^. Therefore, under stimuli coming from the tumoral niche - composed of mesenchymal cells, tumor-infiltrating immune cells (TIIC), endothelial cells, extracellular matrix, and inflammatory mediators - the stemness phenotype can be acquired also by non-CSCs. In particular, cancer cells that can enter a reversible drug-tolerant “persister” state in response to treatment have been described^[65]^; this population is made of cycling and non-cycling persisters which arise from different cell lineages with distinct transcriptional and metabolic programs. Upregulation of antioxidant gene programs and a metabolic shift to fatty acid oxidation has been associated with persister proliferative capacity across multiple cancer types, including CRC. Notably, using different inhibitors to impede oxidative stress or metabolic reprogramming led to a significant reduction in the fraction of cycling persisters^[66]^.

In CRC it has been shown that chemotherapy enriches for cells with a CSC phenotype. However, a pivotal role for a full definition of functional stem cell is concomitantly played by tumor microenvironment (TME) properties and in particular by osteopontin - a multifunctional protein that can also act as a cytokine - produced by key cancer-associated fibroblasts (CAFs)^[67]^. Recently, an escape mechanism leading to tumor re-growth after 5-FU treatment has been identified in a p53-mediated activation of the WNT/beta-catenin signaling, a pivotal pathway to sustain CRC CSCs. Accordingly, the addition of a WNT inhibitor to 5-FU effectively suppressed the CSCs and reduced tumor re-growth after discontinuing the treatment^[68]^. Also, CRC CSCs can escape immune surveillance by avoiding recognition by the innate immune system and shape the TME through the release of exosomes, cytokines, and chemokines to generate an immunosuppressive niche that facilitates cancer progression^[69]^.

As mentioned above, TME cues from the tumoral niche can support drug resistance. Important players in this scenario are CAFs, which represent an essential component of tumoral stroma and produce several cytokines acting on tumor cells. Beyond osteopontin, another multifunctional cytokine released by CAFs is transforming growth factor beta (TGFB) that can act synergistically with hypoxia-induced tumor cell-expressed HIF1A to sustain 5-FU/oxaliplatin resistance via activation of the hedgehog pathway, as demonstrated by *in vitro *and *in vivo *experiments using patient-derived cells^[70]^. Interestingly, 5-FU-induced TGFB production occurs also in CRC cells as a mechanism of resistance and targeting TGFBRI restores the sensitivity of drug-resistant cells to 5-FU toxicity^[71]^. In addition, the 5-FU-elicited TGFB1 protective action can also be abolished by the use of BTK inhibitors^[51]^. Other than through cytokine release, CAFs can communicate with the cancer cell directly, by transferring exosomes. For example, it has been shown that exosomal transfer of miR-92a-3p to CRC cells activates the WNT/beta-catenin pathway and inhibits mitochondrial apoptosis, and contributes to cell stemness, epithelial-to-mesenchymal transition, metastasis, and resistance to 5-FU/oxaliplatin treatment^[72]^. Also, exosomal transfer of lncRNA H19 has been found to promote the stemness and chemo-resistance of CRC via activation of the WNT/beta-catenin pathway due to its action as a competing endogenous RNA sponge for miR-141^[73]^. Notably, these data further indicate WNT/beta-catenin pathway as a potentially actionable target to overcome chemotherapy resistance.

Exosomes can also be exchanged by cancer cells themselves either in normal conditions or upon chemotherapy. For example, it has been shown that miR-21 is significantly upregulated in exosomes purified from CRC cell lines and that adding them to the cells induces resistance against 5-FU through the downregulation of PDCD4^[74]^. Exosomes derived from 5-FU-resistant cells instead are enriched in growth/differentiation factor 15 (GDF15) and dipeptidyl peptidase IV (DPP4), both of which proved to be potent inducers of angiogenesis^[75,76]^. Accordingly, 5-FU-resistant CRC cells showed high microvascular density *in vivo*^[75]^. Taken together these data indicate that GDF15 and DPP4 may be novel targets for inhibiting angiogenesis in 5-FU-resistant colon cancers.

Finally, in the tumoral niche, different types of TIICs are present that interact with cancer cells and the other components of the niche through cytokine production, eventually altering tumor growth and its response to drug therapy^[69,77]^. TIICs in the TME have dual functions in cancer progression: TIIC-related inflammation facilitates tumorigenesis, but TIICs also harbor antitumor properties when appropriately activated. Cancer cell-secreted factors hijack TIIC functions to promote tumor development and metastasis and to suppress immune recognition^[69]^. Accordingly, one of the most important progress in cancer treatment has been the recent addition to chemotherapy of the so-called immune checkpoint (ICIs) inhibitors that act by interrupting the immunosuppressive signals within the TME and reactivating antitumor immunity^[78]^.

### The gut microbiota

Gut microbiota seems to be implicated in chemotherapy efficacy through numerous mechanisms [Table 3], including xenometabolism, immune interactions, and altered community structure. Evidence suggests a potential relationship between the presence of *Fusobacterium nucleatum* and resistance to 5-FU and oxaliplatin chemotherapy and no response to immunotherapy. For example, Yu *et al*.^[79]^ reported that *F. nucleatum* was abundant in CRC tissues in patients with recurrence post-chemotherapy and associated with clinicopathological characteristics. Mechanistically, F. nucleatum targeted TLR4 and MYD88 innate immune signaling and specific microRNAs to activate the autophagy pathway and support drug resistance^[79]^. Other mechanisms by which *F. nucleatum* confers resistance to chemotherapy have been reported, such as the upregulation of BIRC3 expression (a member of the inhibitor of apoptosis family, IAPs)^[80] ^and the activation of the WNT/beta-catenin pathway via a TLR4/P-PAK1 cascade^[81]^. In an animal model of CRC, the intratumor presence of bacteria belonging to the Gammaproteobacteria class and producing the long form of the bacterial enzyme cytidine deaminase, which mediates gemcitabine deamination, conferred resistance to gemcitabine; accordingly, the elimination of bacteria by ciprofloxacin treatment restored the response to chemotherapy. In addition, the presence of *Mycoplasma hyorhinis* determined resistance to gemcitabine^[82]^. At variance, responses to oxaliplatin were reduced in efficacy in tumor-bearing mice that lacked microbiota^[83]^ indicating that the microbial population resident in the gut can affect both sensitivity and resistance to chemotherapy, being these effects modulated by different bacteria.

**Table 3:** The gut microbiota and the response to chemotherapy/immunotherapy

**Bacterium**	**Correlation with drug resistance**	**Mechanism**	**Ref.**
***Fusobacterium nucleatum***	Abundant in CRC tissues of patients with recurrence post-chemotherapy and associated with clinicopathological characteristics	Targeting of TLR4 and MYD88 innate immune signaling and targeting specific microRNAs to activate the autophagy pathway	[79]
		Upregulation of BIRC3 expression	[80]
		Activation of the WNT/beta-catenin pathway via a TLR4/P-PAK1 cascade	[81]
Gammaproteobacteria ***Mycoplasma hyorhinis***	Resistance to gemcitabine reverted by eliminating bacteria by ciprofloxacin treatment	Production of the long form of the bacterial enzyme cytidine deaminase which mediates gemcitabine deamination	[82]
Whole microbiota	Reduction of the response to oxaliplatin in mice lacking microbiota		[83]
***Bacteroides thetaiotaomicron*** ***Bacteroides fragilis***	Their introduction in SPF mice models of CRC restored the response to anti-CTLA-4 treatment, absent in germ-free or antibiotic treated mice		[147]

SPF: Specific pathogen-free.

Finally, it has been recently shown that commensal bacteria can produce extracellular vesicles (EVs) able to deliver to the human cells bacterial RNA molecules with gene expression regulatory ability, thus suggesting that they might potentially regulate the expression of genes involved in drug resistance. The communication between commensal bacteria and host cells have been shown to be bi-directional suggesting a reciprocal regulation of gene expression and accordingly, biological cell function. An example of this communication having an impact in the development of drug resistance is offered by the previously mentioned study by Yu *et al*.^[79]^, where resistance to 5-FU and oxaliplatin has been shown to be mediated by *F. nucleatus*-induced autophagy through the TLR4/MYD88-mediated downregulation of miR-18a* and miR-4802. Conversely also intestinal epithelial cells exerted a reciprocal effect onto *F. nucleatus* by controlling its growth through the delivery of specific miRNAs, such as hsa-miR-515-5p. In CRC patients, altered expression of miR-515-5p might thus affect *F. nucleatus* proliferation and consequent response to chemotherapeutic drugs^[79]^. EV-mediated intercellular communication between bacteria and cancer cells seems therefore to be another potential mechanism playing a role in determining the efficacy of cancer therapy and worthy of increasing attention.

## RESISTANCE TO TARGETED THERAPY

According to ASCO guidelines, chemotherapy can be possibly integrated with anti-angiogenic agents such as bevacizumab or aflibercept (first-line and second-line treatment, for advanced CRC) and regorafenib (in patients with mCRC who have already received chemotherapy and other targeted therapies). In selected cases, chemotherapy may also be integrated with the addition of targeted therapy. For example, anti-EGFR (anti-epidermal growth factor receptor) monoclonal antibodies (e.g., cetuximab and panitumumab) are indicated only for *RAS*- and *BRAF*-wild type (wt) tumors since the mutation of these two genes confer resistance to the EGFR blockade. In addition, a combination using the BRAF inhibitor encorafenib and cetuximab may be used to treat patients with *BRAF*-mutant (mut) mCRC who have received at least one previous treatment. In the last decade, several tumor-expressed “immune checkpoints”, immunosuppressive molecules blocking antitumor immunity, have been identified and “checkpoint inhibitors” have been developed and entered the clinic. Among them are antibodies against programmed cell PD-1 or its ligand PD-L1 and cytotoxic T lymphocyte-associated protein 4 (CTLA-4). So far, immunotherapy has a limited use in CRC since PD-1 targeting monoclonal antibodies can be used only in selected cases of mCRCs bearing defects along the DNA damage response pathways. For example, pembrolizumab is used for the therapy of mCRCs that are MSI-H or dMMR. In contrast, nivolumab can be administered to patients with dMMR or MSI-H mCRC that has grown or spread after treatment with chemotherapy, either alone or in combination with ipilimumab (anti-CTLA-4 monoclonal antibody). Notably, the FDA has issued its approval for pembrolizumab as the first-line treatment of patients with dMMR/MSI-H mCRC in June 2020, even though neither the Japanese Pharmaceuticals and Medical Devices Agency nor the European Medicines Agency has yet approved pembrolizumab as the frontline regimen^[84]^.

To expand the range of possible combinations of precision drugs to add to chemotherapy several efforts have been made to better stratify patients and to understand the basis of resistance/insensitivity to targeted therapy.

### Resistance to anti-EGFR blockade

**Figure 2:**
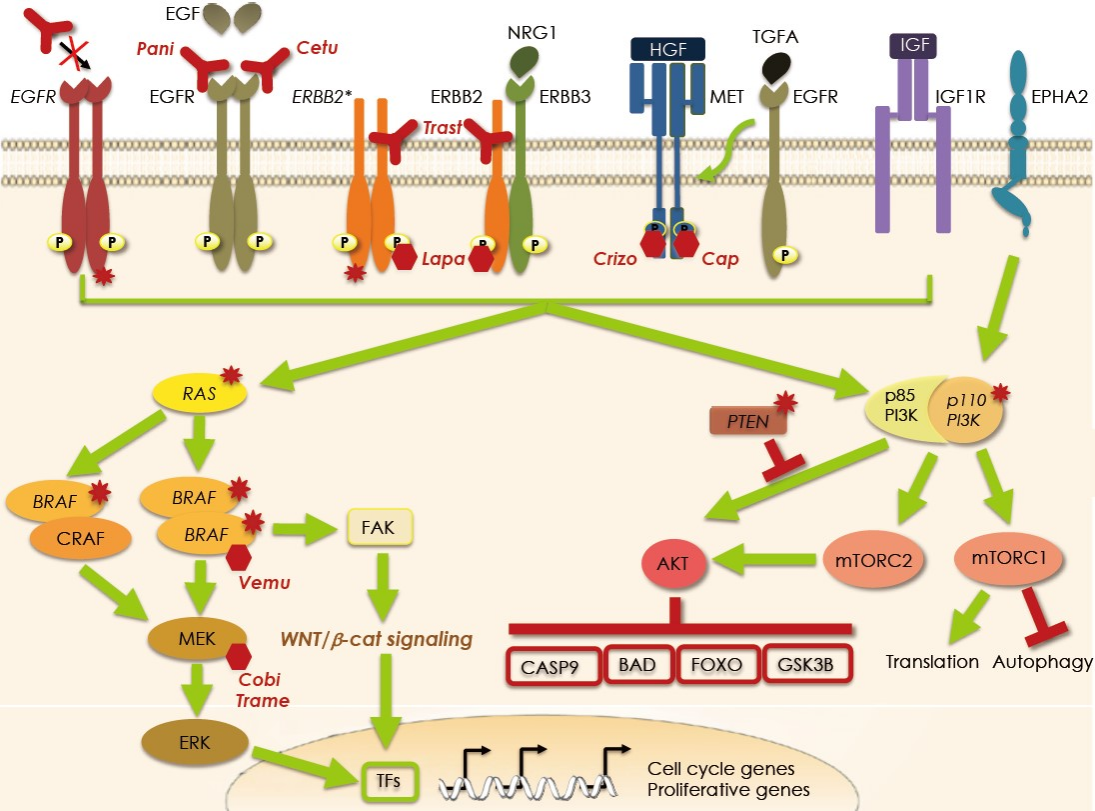
Mechanisms of resistance to targeted therapy against EGFR and mutated *BRAF*. Intrinsic resistance to EGFR blockade by panitumumab (Pani) and cetuximab (Cetu) occurs in case of activating mutations in the *EGFR* and its downstream effectors such as RAS, BRAF, and PIK3CA and when there is loss of *PTEN* either due to truncation or deletion. Acquired resistance stems from stimulation of collateral pathways impinging on the same downstream signaling activated by EGFR (i.e., the RAS/MAPK and the PIK3/AKT pathways). Hyperactivation of these pathways may be due to overexpression or mutation of *ERBB2*, heterodimerization of ERBB2 and ERBB3, engagement of the EGFR by TGFA which in turn activates MET, MET overexpression, EPHA2 overexpression, or IGF1R engagement. Strategies to overcome the insensitivity to EGFR blockade are directed at the inhibition of the collateral signaling such as using ERBB2 blocking agents trastuzumab (Trast) and lapatinib (Lapa) and the MET inhibitors crizotinib (Crizo) and capmatinib (Cap). Resistance to the mutated BRAF inhibitor vemurafenib (Vemu) originates from the shifting in the choice of the dimerization companion. In the resistant cells instead of mutated BRAF homodimers, mutated BRAF/CRAF heterodimers are formed which are insensitive to the inhibitor and thus MAPK activation is refueled. To prevent hetodimerization a novel inhibitor, that specifically bind mutated BRAF homodimers has been developed. Another route of escape to mutant BRAF blockade has been described where activation of the WNT/β-catenin signaling is triggered via FAK. Both MAPK and β-catenin signaling eventually activate a series of transcription factors (TFs) responsible for the transcription of proliferative genes. Currently, to prevent resistance to BRAF inhibition, a vertical blockade of the pathway is being tested in clinical trials using vemurafenib in combination with a MEK inhibitor such as cobimetinib (Cobi) or trametinib (Trame). Mutated proteins are indicated by the red stars and the italicized name.

Perhaps the best studied so far are the mechanisms and the determinants underlying the resistance to anti-EGFR drugs [Figure 2], given that they have been the first targeted drugs employed for the treatment of CRC, and are so far the most used [Tables 4 and 5]. The main genetic defects hampering the response to EGFR blockade are activating mutations leading to constitutive activation of signaling pathways downstream of EGFR such as mutations in *KRAS *(30%-40%), *NRAS *(4%), *BRAF *(7%-15%), or *PIK3CA* (17%-30%) genes and *PTEN* deletion or truncation (7%) which altogether account for unresponsiveness in around 70% of cases^[85,86]^. It has been reported that in approximately 50% of *RAS-*wt patients, sensitivity to EGFR blockade is lost because of secondary occurring mutations^[87]^. In a small fraction of the remaining resistant tumors two important biomarkers are emerging. Bardelli *et al*.^[88]^ initially reported that certain patients harboring *RAS-*wt gene and resistant to anti-EGFR therapy presented an amplification of the *MET* proto-oncogene that proved to be responsible for *de novo* and acquired resistance to anti-EGFR therapy. In fact, functional studies showed that MET activation conferred resistance to anti-EGFR therapy both *in vitro* and *in vivo *and could be overcome using MET inhibitors^[88]^. Remarkably, examining circulating tumor DNA, it was possible to find MET amplification even before the recurrence became clinically evident. Much experimental evidence further supported the benefit of targeting MET in anti-EGFR-resistant CRCs and explored the mechanisms driven by MET. Among them, acquisition of cetuximab resistance was shown to be mediated by TGFA overexpression which in turn induced EGFR-MET interaction^[89]^. Also, SRC activation promoted cetuximab resistance by directly interacting with MET; accordingly, pretreatment with SRC inhibitors abolished cetuximab-mediated MET activation and rendered CRC cells sensitive to cetuximab^[90]^. Interestingly, concurrent inhibition of MET and SRC decreased viability and enhanced apoptosis in both *RAS*-wt and *RAS*-mut CRC cells. Therefore, combined inhibition of MET and SRC may be a promising strategy for treating of CRC, independent of anti-EGFR resistance^[91]^. In addition, MET inhibition, by enhancing the formation of DNA double-strand breaks and possibly alleviating tumor hypoxia, has been found to sensitize CRC cells also to irradiation^[92]^, further expanding the actionability of MET in CRCs.

**Table 4:** Resistance to anti-EGFR blockade

**Treatment**	**Mutational background**	**Mechanism**	**Ref.**
Cetuximab	Activating mutations in *KRAS*, *NRAS*, *BRAF* or *PIK3CA* genes and *PTEN* deletion, truncation, or epigenetic silencing	Constitutive activation of signaling pathways downstream of EGFR	[85,86]
Cetuximab Panitumumab	*MET* amplification in wt*KRAS *setting	Activation of signaling pathways converging onto PIK3CA and MAPK; resistance bypassed using MET inhibitors	[88]
Cetuximab		TGFA overexpression which in turn induced EGFR-MET interaction	[89]
Cetuximab		Cetuximab-mediated MET activation via interaction with SRC and abolished by SRC inhibitors	[90]
Cetuximab	*ERBB2* amplification /mutations in quadruple (*KRAS*, *NRAS*, *BRAF*, and *PIK3CA*) wt setting	Constitutive activation of signaling pathways downstream of the receptor; resistance overcome by addition of anti-ERBB2 agents	[97,98]
Cetuximab	*ERBB2* amplification and heregulin overexpression	ERBB2/ERBB3 dimerization; resistance bypassed by silencing ERBB2 and depleting heregulin	[101]
Intrinsic resistance to anti-EGFR agents	*ERBB2 *activating mutations in* KRAS-*wt mCRCs	Constitutive activation of signaling pathways downstream of the receptor; resistance bypassed by dual targeting of ERBB2 via trastuzumab and lapatinib	[103]
Cetuximab+Refametinib	*KRAS-*mut	PI3K-AKT pathway activation ensuing from autocrine loops and heterodimerization of multiple receptors (ERBB2, ERBB3, and IGF1R)	[112]
Cetuximab+FOLFIRI	*RAS*-wt mCRC	High levels of EPHA2 significantly correlated with worse PSF and increased progression rate in a cohort of patients; primary and acquired resistance to cetuximab reverted in in *in vitro* and *in vivo* models by adding a specific EPHA2 inhibitor	[114]

FOLFIRI: Folinic acid + 5-fluorouracil + irinotecan; PSF: progression free survival.

**Table 5:** Strategies to overcome resistance to EGFR inhibition

**Drug(s)**	**Mutational background**	**Mechanism**	**Ref.**
Cetuximab + Crizotinib	*MET* amplification	Double targeting of EGFR and MET	[88]
Cetuximab + PHA665752	Overexpression of TGF-α	Double targeting of EGFR and MET	[89]
Cetuximab + PHA665752 Cetuximab + Dasatinib	*RAS*-wt; *MET* activation	Double targeting of EGFR and MET Double targeting of EGFR and SRC	[90]
Cetuximab + Lapatinib Cetuximab + Pertuzumab	*ERBB2 *amplification	Double targeting of EGFR and ERBB2	[97]
Trastuzumab + Neratinib Trastuzumab + Afatinib	*ERBB2 *amplification or mutation	Double targeting of ERBB2	[98]
Trastuzumab + Lapatinib (clinical trial)	*ERBB2* overexpression; *RAS*-wt	Double targeting of ERBB2	[103]
Cetuximab + ALW-II-41-27	EPHA2 overexpression	Double targeting of EGFR and EPHA2	[114]

PHA665752: Small-molecule inhibitor of MET; ALW-II-41-27: EPH family tyrosine kinase inhibitor.

Despite experimental efficacy, the clinical trials performed so far did not confirm the expectations. In a phase 2 study the addition of the specific MET inhibitor tivantinib to cetuximab allowed only 10% of MET-high, *KRAS*-wt mCRC patients to achieve objective response, even though in a difficult-to-treat setting (tumor progression on cetuximab or panitumumab after a first-line chemotherapy)^[93]^.

In another phase 1/2 study addition of tivantinib to cetuximab + irinotecan was tested in *KRAS-*wt patients previously treated but who progressed or presented with mCRC. In these cases, the combination did not significantly improve progression-free survival (PFS), even though subgroup analyses trended in favor of tivantinib in patients with MET-high tumors, PTEN-low tumors, or those pretreated with oxaliplatin^[94]^. One possible explanation for these disappointing clinical results might be that clinical efficacy has been obscured by a lack of standardization in MET assessment for patient stratification. Accordingly, a very recent paper indicated that subtyping of MET expression may be required to identify MET-addicted malignancies in CRC patients who will truly benefit from MET inhibition^[95]^.‬‬‬‬‬‬‬‬

In 2011 *ERBB2* gene amplification was reported in 7% of patients with CRC^[96]^. Subsequently, *ERBB2* amplification or somatic mutations have been described by several studies, with highly different reported rates of positivity (up to 50%) likely due to several factors such as cohort heterogeneity, a small study population, antibody clone selection, staining platform, and different scoring system. Using a molecularly annotated platform of 85 mCRC patient-derived xenografts, Bertotti *et al*.^[97]^ identified *ERBB2 *amplification as an actionable liability in cetuximab-resistant CRCs harboring wt *KRAS*/*NRAS*/*BRAF*/*PIK3CA.* Moreover, they showed that the addition of anti-ERBB2 agents to anti-EGFR monoclonal antibodies overcame resistance and inhibited tumor growth^[97]^. The same group also showed that tumor growth was reduced by single targeting of ERBB2 in cetuximab-resistant, quadruple (i.e., *KRAS*, *NRAS*, *BRAF*, and *PIK3CA*) wt CRC patient derived xenografts with *ERBB2* mutations. However, only double targeting of EGFR and ERBB2 led to durable tumor regression^[98]^.

Later, the role of *ERBB2* amplification/mutation in predicting response to anti-EGFR treatment was confirmed by different groups. Cremolini *et al*.^[99]^, conducted a prospective, case-control study to demonstrate the negative predictive impact of a panel of rare genomic alteration including *ERRB2* amplification/activating mutations, *MET* amplification, *ROS1/NTRK1-3/RET* rearrangements, and* PIK3CA* exon 20, *PTEN*, and *ALK* mutations. Of the resistant cases, 51.1% were associated with one of these candidate molecular alterations, *ERBB2* amplification being the most frequent (14.9%), followed by *MET* amplification (8.5%)^[99]^. A retrospective study performed in *RAS/BRAF*-wt mCRC patients reported that anti-EFGR therapy allowed a PFS significantly longer in patients without *ERBB2* amplification than in those bearing the alteration^[100]^. Shorter PFS and OS were demonstrated also in cetuximab-treated CRC patients with *ERBB2*-amplified tumors and higher serum heregulin levels. In addition, *in vitro* experiments proved that silencing ERBB2 overexpression, as well as depleting heregulin - thus disrupting of ERBB2/ERBB3 heterodimerization - restored the response to cetuximab^[101]^. Notably, ERBB2 amplification could be identified non only in tissues but also in circulating tumor DNA of CRC patients non-responsive to anti-EGFR therapy^[102]^. Sartore-Bianchi *et al*.^[103]^, demonstrated that *ERBB2 *activating mutations in *KRAS*-wt mCRCs predicted intrinsic resistance to anti-EGFR agents that could be bypassed by combining dual targeting ERBB2 via trastuzumab (anti-ERBB2 monoclonal antibody) and lapatinib (dual EGFR and ERBB2 tyrosine kinase inhibitor). A phase 2 basket trial recently obtained an overall response rate of 32% when using the dual targeting approach (pertuzumab and trastuzumab) in pretreated *ERBB2*-amplified mCRC patients^[104]^. In addition, two other phase 2 clinical trials also identified ERBB2 as a druggable target in refractory *KRAS*-wt mCRCs with *ERBB2* amplification^[105,106]^, and ERBB2-targeted combinations have been recently included in international guidelines for mCRC^[107]^.

Besides the above-mentioned clinical studies, several trials are currently undergoing where ERRB2-targeted antibody-drug conjugates (i.e., TDM-1, DS-8201, A166, ZW25, and ZW49) and novel tyrosine kinase inhibitors (i.e., tucatinib, sapitinib, neratinib, pyrotinib, poziotinib, and ceralasertib) are being tested alone or in different combinations^[108]^. A recent study pinpointed that an optimal combination may require triple targeting. Mangiapane *et al*.^[109]^, using 60 CRC specimens obtained a collection of CSC-derived spheres which was then used to perform preclinical experiments to validate different therapeutic options. They found that high expression levels of ERBB2 occurred in spheres showing hyperactive PI3K/AKT pathway and proved that triple targeting of ERBB2, MEK and PI3K induces CSC death and regression of anti-EGFR-resistant tumor xenografts, including those carrying *KRAS* and *PIK3CA* mutation^[109]^.

Even though MAPK deregulation plays a pivotal role in both intrinsic and secondary resistance to anti-EGFR agents, other mechanisms (sometimes co-occurring) contribute or can account for secondary resistance, due to CRC molecular heterogeneity^[110]^. For example, stimulation of EGFR results not only in the RAS/RAF/MAPK pathway but also in the PTEN-PI3K-AKT cascade activation^[111]^.

Recently, Vitiello *et al*.^[112]^ used four different *KRAS-*mut CRC cell lines rendered resistant to a combination of cetuximab and refametinib (a selective MEK-inhibitor) and demonstrated that PI3K-AKT pathway activation acts as an escape mechanism in this setting. They showed that autocrine loops and heterodimerization of multiple receptors lead to the activation of several receptor tyrosine kinase, such as ERBB2, ERBB3 and IGF1R, whose signaling onto the PI3K/AKT pathway allows to bypass the block imposed by the double targeting of EGFR and MEK^[112]^. The effect on the response to cetuximab in the setting of the three most common *AKT1* activating mutations found in CRC patients (i.e., E17K, E49K, and L52R) was recently investigated by overexpressing them in a cetuximab-sensitive *KRAS-*wt CRC cell line. Interestingly, all of them impaired the cytotoxic response not only toward cetuximab but also to chemotherapy. In addition, also the most common mutations of *CTNNB1* (i.e., T41A, S45F, and S33P) were found to sustain resistance to the same agents^[113]^. These data indicate that possible therapeutic strategies to counteract the resistance to EGFR-targeted therapy would therefore be the use of inhibitors of the PI3K/AKT pathway. Notably, trials to test AKT and PI3K inhibitors for CRC are currently undergoing.

A novel predictive biomarker of resistance and a potential therapeutic target for improving the response to anti-EGFR agents has recently been identified in EPHA2. Studying a cohort of 82 *RAS*-wt mCRC patients treated with FOLFIRI + cetuximab, Martini *et al*.^[114]^ found that 62.6% of patients expressed the tyrosine kinase receptor and that high levels of EPHA2 significantly correlated with worse PFS and increased progression rate. In *in vitro* models a specific EPHA2 inhibitor reverted primary and acquired resistance to cetuximab causing cell growth inhibition, inducing apoptosis and cell-cycle G1-G2 arrest. In xenografts experiments the treatment with the inhibitor upon progression with cetuximab significantly inhibited tumor growth^[114]^.

### Resistance to BRAF inhibitors

The BRAF kinase, operating downstream of RAS along the pathway leading to ERK activation, is considered an oncogenic driver in CRC and its mutation arises in 7%-15% of patients with mCRC. Over 95% of *BRAF* mutations in mCRC occur in codon 600 (V600E) whereas non-V600E *BRAF* mutations occur only in about 2% of patients and define a clinically distinct subtype with a better prognosis. In general, patients with *BRAF*-mut CRC have impaired survival not only in the metastatic setting but also in non-metastatic disease, as compared with patients with *BRAF*-wt CRC, and are resistant to chemotherapy. In fact, the median OS is 4 to 6 months after failure of initial therapy. Currently, SOC is an aggressive strategy involving triplet chemotherapy and anti-VEGF agents, but no specific tailored therapeutic approach has been standardized since no optimal combinations have been reported yet. In fact, at variance with what was observed for melanoma, vemurafenib - the first specific BRAF^V600E^ inhibitor tested - showed modest clinical activity in mCRC patients with *BRAF*-mut tumors^[115]^. In addition, no responses were observed in the 10 patients with *BRAF*^V600E^ mCRC enrolled in a phase 2 basket trial^[116]^. Overall, only 5% of patients with *BRAF*^V600E^ CRC respond to BRAF inhibitors^[117]^ indicating a very high level of intrinsic resistance to its blockade [Tables 6 and 7]. The reason for the unresponsiveness to vemurafenib was uncovered in two key studies where a paradoxical effect ensuing from BRAF inhibition was shown in *BRAF*^V600E^ CRC models. BRAF^V600E^ can signal as RAS-independent monomers or dimers - depending on levels of RAS activation in the tumor - but predominantly exists as a drug-sensitive monomer. Vemurafenib selectively inhibits BRAF monomers. It was discovered that BRAF^V600E^ inhibition suppressed ERK-mediated negative feedback on EGFR activity, thus leading to EGFR-mediated reactivation of MAPK signaling, due to the formation of active RAF and CRAF protein dimers^[118,119]^. Recently, PLX8394, a novel experimental BRAF inhibitor has been reported to inhibit ERK signaling by specifically disrupting BRAF-containing dimers, thus acting on tumors driven by dimeric BRAF mutants and opening a new possibility to overcome resistance to classic BRAF inhibitors^[120]^. Interestingly, few possible targets for combinatorial treatment have been experimentally identified in *BRAF*^V600E^ CRCs. For example, it has been shown that in this setting anti-apoptotic MCL1 is upregulated due to ERK-mediated phosphorylation which results in stabilization of the protein. Even though MEK inhibitor cobimetinib suppressed MCL-1 phosphorylation to a greater extent than did vemurafenib, it resulted in only mildly cytotoxic effects. At variance co-targeting MEK and MCL-1 (either via silencing or via a small-molecule antagonist) significantly increased cytotoxicity *in vitro* and reduced tumor growth *in vivo*^[121]^. Chen *et al*.^[122]^ demonstrated that in *BRAF*^V600E^ CRC cell lines BRAF inhibitors upregulated the WNT/beta-catenin pathway through activating FAK independently of EGFR and MEK signaling [Figure 2]. Accordingly, combined inhibition of BRAF/WNT pathways or BRAF/FAK pathways exerted strong synergistic antitumor both *in vitro* and *in vivo*^[122]^. Recently, another mechanism of resistance to vemurafenib has been identified in the increased abundance and activity of nucleophosmin (NPM1), a protein that regulate centrosome duplication and histone assembly and that is induced by the inhibitor in resistant cells. Accordingly, pharmacological inhibition of NPM1 effectively restored the sensitivity of vemurafenib-resistant *BRAF-*mut CRC cell lines^[123]^.

**Table 6:** Resistance to BRAF inhibitors

**BRAFi**	**Mutational background**	**Mechanism**	**Ref.**
Vemurafenib	BRAF^V600E^	Only BRAF monomers inhibited by vemurafenib; EGFR-mediated reactivation of MAPK signaling, due to the formation of active RAF and CRAF protein dimers	[118,119]
Vemurafenib	BRAF^V600E^	Upregulation of Wnt/β-catenin pathway through activation of FAK	[122]
Vemurafenib	BRAF^V600E^	Upregulation of NPM1	[123]

**Table 7:** Strategies to overcome resistance to BRAF inhibitors

**Drug(s)**	**Mutational background**	**Mechanism**	**Ref.**
PLX8394	BRAF^V600E^	Specific inhibition of BRAF dimers; prevent EGFR-mediated reactivation of MAPK signaling	[120]
Cobimetinib + A-1210477	BRAF^V600E^	Double targeting of MEK and MCL-1	[121]
Vemurafenib + PF-562271 Vemurafenib + ICG-001 Vemurafenib + PF-562271 + trametinib Vemurafenib + ICG-001 + trametinib	BRAF^V600E^	Double targeting of BRAF^V600E^ and FAK Double targeting of BRAF^V600E^ and β-catenin Triple targeting of BRAF^V600E^, FAK, and MEK Triple targeting of BRAF^V600E^, β-cat, and MEK	[122]
Vemurafenib + NSC348884	BRAF^V600E^	Double targeting of BRAF^V600E^ and NPM1	[123]
Encorafenib + Cetuximab (clinical trial)	BRAF^V600E^	Double targeting of BRAF^V600E^ and EGFR	[136]

PLX8394: RAF inhibitor; A-1210477: elective MCL-1 inhibitor; PF-562271: FAK inhibitor; ICG-001: beta-catenin inhibitor; NSC348884: nucleophosmin (NPM1) inhibitor.

Given the EGFR reactivation observed in BRAF inhibitor-treated CRC models, dual targeting has been tested. Adding cetuximab to vemurafenib in xenografts experiments resulted in increased antitumor activity and improved survival^[124]^ and combined administration of BRAF and EGFR inhibitors induces tumor regression in most patients^[116,125-127]^. However, within 6 months, resistance inevitably occurs. Analyzing matched tumor tissues obtained from eight patients before treatment and at the time of disease progression after therapy, Yaeger *et al*.^[128]^ found that in resistant tumors new alterations occurred in genes that encode components of the RAS/RAF pathway (activating mutations of *KRAS* or *NRAS* or amplification of *NRAS*-wt, *KRAS*-wt, or *BRAF*^V600E^*).* They then overexpressed *NRAS*-wt or *KRAS* wt in *BRAF*^V600E^ CRC cell lines sensitive to vemurafenib/cetuximab treatment, demonstrating that the increase in either of the two was sufficient to induce the presence of RAF and CRAF protein dimers (absent in sensitive cells) thus rendering the cells resistant to the combinatorial therapy^[128]^. Therefore, it would be interesting to verify in this setting the effect of the inhibitor PLX8394 that acts by disrupting dimers^[120]^.

Preclinical and clinical data obtained in *BRAF*^V600E^ melanoma models and patients indicated that simultaneous and vertical targeting of more than one node along the MAPK/ERK pathway, such as BRAF and MEK, could bypass the resistance to BRAF inhibitors^[129,130]^ and delay the onset of relapse. However, also this combination did not live up to expectations in *BRAF*^V600E ^CRCs. In fact, combination treatment with BRAF plus MEK inhibitors, dabrafenib and trametinib, led to a median PFS of 3.5 months, with 56% of patients achieving stable disease, 9.3% partial response, and only 2.3% complete response^[131]^. It has been recently shown that the combination of the BRAF inhibitor dabrafenib and the MEK inhibitor trametinib triggered a massive apoptotic response only in *BRAF*^V600E ^CRC cells harboring also *TERT* promoter mutations but not in *BRAF*^V600E^ cells lacking* TERT* mutations. Accordingly, the combination of inhibitors nearly completely abolished the growth of xenografted tumors *BRAF*^V600E^/*TERT*-mut but had little effect on tumors harboring only *BRAF*^V600E^. Notably, after the treatment was discontinued, doubly mutated tumors remained barely measurable whereas *BRAF*^V600E^ tumors regrew rapidly^[132]^. These findings suggest that double testing for *BRAF*^V600E^ and *TERT* mutations may stratify patients for combinatorial therapy with BRAF plus MEK inhibitors.

Recent clinical trials investigated the benefit of targeting different nodes along the MAPK pathway in *BRAF-*mut mCRC^[133-135]^. BEACON III was a three-arm phase 3 study that enrolled patients with *BRAF*^V600E^ mCRC tumors who progressed on one or two prior regimens. In this setting the safety and efficacy of encorafenib (BRAF inhibitor) and cetuximab with or without binimetinib (MEK inhibitor) were evaluated and compared with the control arm with SOC therapy (irinotecan/cetuximab or FOLFIRI/cetuximab). Both triplet and doublet therapy induced an overall response rate that was 10 times more than in the control arm. In addition, PFS tripled and mean OS doubled for the doublet and triplet arms *vs*. control arm. Little difference was observed between doublet and triplet arms^[136]^. Based on these results, on April 2020, the FDA approved encorafenib in combination with cetuximab for mCRCs with a *BRAF*^V600E^ mutation^[137]^.

A final note, not directly related to resistance but pointing to alternative actionable targets in *BRAF*^V600E^ CRCs, should be made about immunotherapy. As previously mentioned, therapy with ICIs is limited to mCRCs bearing defects along DNA damage response pathways. Notably, around 60% of *BRAF*-mut CRCs also are MSI-H, a condition associated with a higher proportion of tumor infiltrating lymphocytes present in the tumor^[138]^. Therefore, it is reasonable to suggest that ICIs could represent the new SOC for this subgroup^[139]^.

### Resistance to anti-angiogenic therapy

The first anti-angiogenic agent entering the clinic for the treatment of mCRC has been bevacizumab, a humanized monoclonal antibody anti-VEGF-A which binds circulating VEGF-A and blocks the interaction with its cell surface receptors (VEGFR1 and 2), thus reducing microvascular growth and inhibiting the blood supply to the tumor tissues^[140]^. In recent years the development of anti-angiogenic agents has increased, leading to the addition to of aflibercept (second-line treatment for advanced CRC) and regorafenib (in patients with mCRC who have already received chemotherapy and other targeted therapies). Aflibercept is a recombinant fusion protein consisting of VEGF-binding portions from the extracellular domains of human VEGFR1 and 2, that are fused to the Fc portion of the human IgG1 immunoglobulin and acts like a “VEGF trap”. Regorafenib is multi-kinase inhibitor targeting angiogenic, stromal, and oncogenic receptor tyrosine kinase that shows strong anti-angiogenic activity due to its dual targeted VEGFR2-TIE2 tyrosine kinase inhibition. In addition, other anti-angiogenic drugs are in preclinical and clinical phase I-III studies^[141]^.

**Figure 3:**
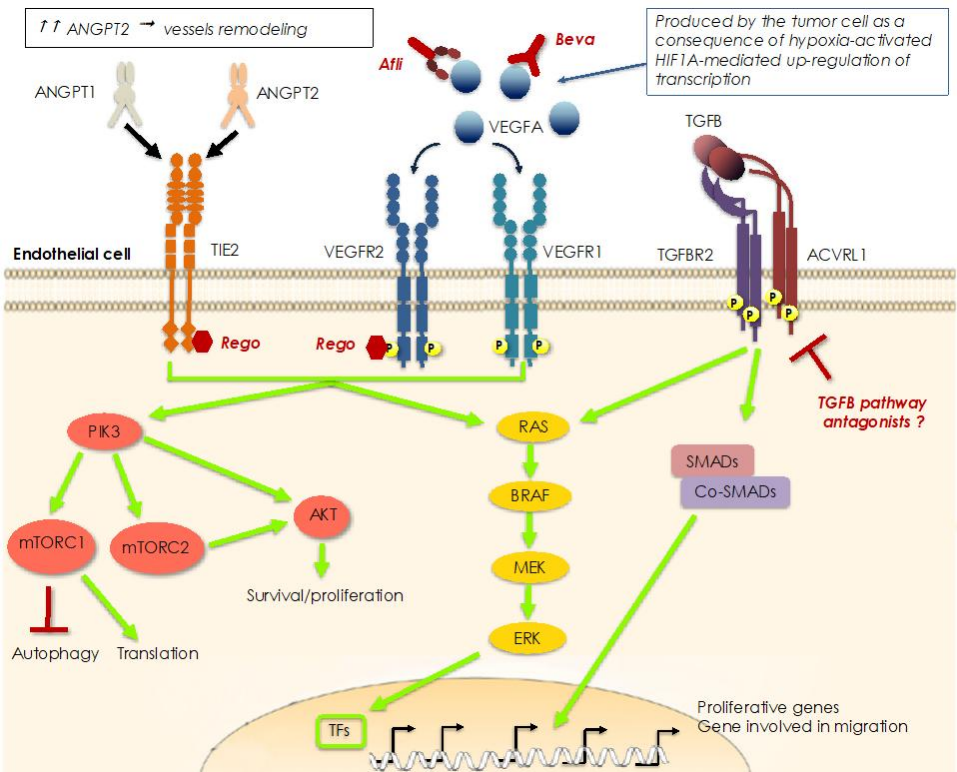
Mechanisms of resistance to anti-angiogenic therapy. Hyperproduction of VEGFA is a consequence of the hypoxic status in the center of the tumor which activates HIF1A, a transcription factor that regulates VEGFA transcription. Overproduction of VEGFA can be blocked by monoclonal antibodies (bevacizumab, Beva) or a VEGF-trap (aflibercept, Afli). Alternatively, the signal can be blocked downstream of the dedicated receptor by small kinase inhibitors like regorafenib (Rego), which can also inhibit the TIE2 receptor, thus imposing a double anti-angiogenic blockade. In fact, both receptors are critical in stimulating the proliferation and migration of the endothelial cells, necessary for the formation of new vessels into and around the tumor mass. In particular, TIE2 receptor is regulated by ANGPT1 and 2, the former inducing signals involved in maturation and stabilization of blood vessels, whereas the latter blocks this signaling and triggers remodeling of vascular sprouts. Overproduction of ANGPT2 can drive a parallel neo-angiogenic signaling when VEGR-driven signaling is impeded by Beva-based therapy. In addition, another parallel neo-angiogenic pathway that can act as an escape route to VEGFA blockade is initiated by TGFB binding to a multimeric receptor formed by a TGFB2 dimer in complex with an activin receptor-like kinase 1 (ACVRL1) dimer.

Despite initial promising preclinical results, anti-angiogenic monotherapies did not lead to many clinical benefits, due to primary or acquired resistance, through activation of alternative mechanisms that support vascularization and tumor growth [Figure 3]. Clinical data show that anti-angiogenesis therapies can prolong PFS but have a limited impact on OS and do not set a permanent cure in CRC^[142]^. This limited clinical significance is likely due to the fact that treatment with antiangiogenic agents causes a discontinuity in the blood vessel network, which leads to a new hypoxic condition with activation of vascular mimicry, alternative pro-angiogenic pathways and recruitment of endothelial cells derived from bone marrow^[143]^.

So far, the mechanisms of resistance to anti-VEGF therapy have not been fully elucidated. The main factor used by cancer cells to adapt to oxygen deprivation is hypoxia-inducible factor (HIF) of which three members are known (i.e., HIF1A, HIF2A, and HIF3A). During hypoxia, HIF1A translocates into the cell nucleus to form the activated HIF1 complex along with HIF1B. Hypoxic contexts induce upregulation of VEGF expression through the upstream transcription factor HIF1A. These factors induce tumors to acquire more angiogenic and invasive potentials, promoting metastasis^[144]^. Preclinical studies and clinical trials suggest that inhibition of a specific growth factor can induce the expression of others^[145]^. For instance, in a phase II study in which mCRC patients were treated with a combination of 5-FU, leucovorin and irinotecan (FOLFIRI) and bevacizumab, several angiogenic factors, including the placental growth factor and hepatocyte growth factor, were found to increase before disease progression^[146]^. Activin receptor-like kinase 1 (ACVRL1) is an emerging target for antiangiogenic therapy; it is a TGFB transmembrane receptor expressed preferentially from proliferating endothelial cells and is crucial to TGFB-mediated angiogenesis. Significantly, VEGF-A and ACVRL1 expression have been shown to regulate angiogenesis. Data support the hypothesis that ACVRL1 expression and function following anti-VEGF/VEGFR inhibitor treatment may contribute to vascular adaptation from the VEGF-driven neovascularization^[147]^. In particular, the TGFB axis may provide an escape pathway for tumor angiogenesis^[148]^. Thus, agents that inhibit TGFB signaling combined with VEGF/VEGFR- therapies may potentiate treatment response.

Moreover, alternative angiogenic pathway can contribute to the escape from anti-VEGF treatment such as the angiopoietin-TIE signaling system. TIE-1 and -2 are specific vascular receptor tyrosine kinase that control vascular permeability and the development and remodeling of blood vessels through angiopoietin-1 (ANGPT1) and angiopoietin-2 (ANGPT2). While ANGPT1 permits maturation or stabilization of blood vessels through TIE2, ANGPT2 blocks this pathway causing remodeling of vascular sprouts subsequent to exposure to VEGF^[149]^. High serum levels of ANGPT2 have been related to poor response to bevacizumab treatment^[150]^, suggesting that ANGPT2 has a critical role in the resistance mechanism against anti-VEGF therapy^[151]^.

In preclinical models, the double block of VEGF and ANGPT2 inhibited revascularization and tumor progression of tumors resistant to anti-VEGF therapy^[152,153]^. Finally, cytokines have also been involved in mediating resistance to anti-angiogenic therapy. IL-17 and its downstream factor, granulocyte colony-stimulating factor (G-CSF), are the leading players of inflammation; it has been reported that a high level of IL-17 induces cells to produce a paracrine network that confers resistance to anti-VEGF therapy. Serum G-CSF levels in CRC patients are higher than those in healthy volunteers and are associated with the stage of tumor progression^[154,155]^.

Interestingly, a novel anti-angiogenic approach that might help in circumventing resistance to anti-angiogenic therapies could be represented by metronomic chemotherapy, a schedule where low doses of chemotherapy are administered chronically. In fact, several studies demonstrated that the administration of 5-FU under metronomic chemotherapy schedule induces an antiangiogenic effect behind the cytotoxic effect^[156]^. In addition, it has been reported a reasonable disease control with minimal side effects and good quality of life in elderly or heavily pre-treated patients under metronomic chemotherapy schedule. Moreover, a phase III randomized trial has shown that maintenance therapy with metronomic capecitabine plus bevacizumab following cycles of conventional treatment with capecitabine + oxaliplatin + bevacizumab significantly improved PFS compared to the observation group with no worsening of quality of life^[157]^.

### Resistance to immune checkpoint inhibitors

**Figure 4:**
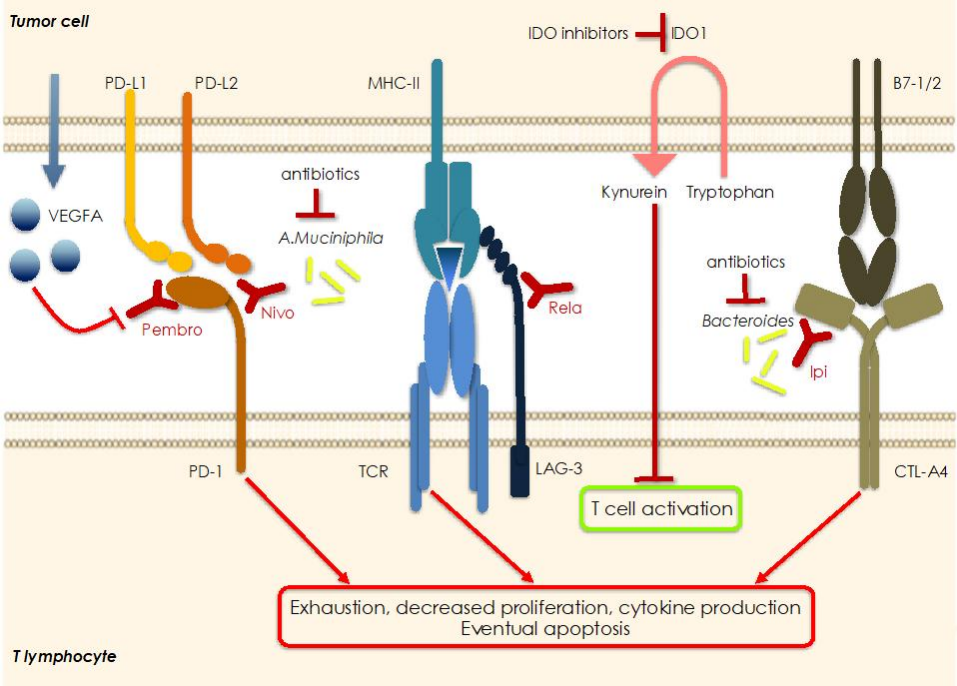
Mechanisms of resistance to immuno-therapy. Cancer cells can express several immunosuppressive molecules that can be blocked using specific inhibitors in order to re-activate the anti-tumor immune response. Often, they are redundant so that the re-activation of one pathway may be compensated by hyperactivation of a different immunosuppressive route so that resistance to therapy is the result. Resistance to anti-PD1 blockade may be due to overproduction and release of VEGFA, as well as by overexpression of IDO1 with consequent increased levels of kynurein in the microenvironment. Specific components of the microbiota are also necessary for the successful blockade of PD-1 and CTL-A4 indicating that antibiotics may represent a resistance factor to ICIs. Finally, concurrent blockade of two or more immune checkpoints has been shown to improve the response and overcome the resistance to ICIs. Pembro: Pembrolizumab; Nivo: nivolumab; Rela: relatlimab; Ipi: ipilumab; IDO1: indoleamine 2, 3-dioxygenase 1.

Compared to pMMR/microsatellite stable CRCs, dMMR/MSI-H CRCs - because of the defects in repairing DNA damage/insertion of microsatellites - present a high mutation burden and produce a plethora of immunogenic neoantigens on the MHC, thus recruiting more tumor infiltrating lymphocytes. However, dMMR/MSI-H CRCs also highly upregulate the expression of immune checkpoints - (i.e., PD-1 ligands, CTLA-4 ligand, LAG-3, and IDO1) that account for the suppression of an active immune response despite the high tumor infiltrating lymphocyte count [Figure 4]^[158]^. Because of this scenario dMMR/MSI-H CRCs have been shown to be sensitive to treatment with ICIs in several phase 2 trials^[84,158-160]^. Notably, the KEYNOTE-177 phase 3 trial compared the effect of anti-PD-1 pembrolizumab *vs*. SOC chemotherapy given as a first line therapy. A significant clinical benefit of pembrolizumab resulting in the doubling of PSF was evident and 83% of pembrolizumab responders were still responding after 2 years or longer, compared to the 35% of the chemotherapy responder. However, 30% of pembrolizumab-treated patients had primary resistance^[161]^ indicating that additional therapeutic strategies are needed in the field of immunotherapy. Interestingly, Checkmate-142 phase 2 clinical trial reported that a double targeting strategy given as a first-line therapy led to significant and clinically meaningful improvements in patients’ outcomes. Specifically, anti-PD-1 nivolumab was administered together with anti-CTLA4 ipilumab, and the PSF rate at 12-month was 77% compared to 55% reported with single PD-1 blockade in the KEYNOTE-177 study^[84]^. At variance with patients with dMMR/MSI-H CRC those with microsatellite stable mCRC had very limited clinical benefit when receiving the combination with CTLA-4 and PD-L1 inhibitors^[160]^. Given that about 95% of mCRC cases are pMMR/microsatellite stable tumors and resistant to ICIs, several efforts are focused on the understanding of the mechanisms of resistance to immunotherapy for the development of new therapeutic approaches. Notably, another promising inhibitor to be added to the combination of ICIs is relatlimab, a monoclonal antibody that binds to LAG-3, a checkpoint molecule that functions to negatively regulate homeostasis of T- and B-cells and NK cells^[162]^. So far, it has been tested in clinical trials for treating melanomas and, interestingly, in a phase 3 trial in patients with newly diagnosed inoperable or metastatic melanomas, its combination with nivolumab doubled PSF^[162]^. In addition, a newly characterized monoclonal anti-LAG-3 antibody resulted in a significant delay in tumor growth when combined with PD-1 blockade in mice transplanted with colorectal cancer cells^[163]^.These results suggest that the addition of anti-LAG-3 monoclonal antibodies might improve the response to currently used ICIs also in CRC. Finally, a brief mention about IDO1 inhibitors is due, given that this enzyme is overexpressed in CRC tissues. IDO1 catalyzes the oxidative cleavage of tryptophan to kynurein, a metabolite responsible for an immunosuppressive environment via the blockade of the activation of effector cells and the stimulation of immunosuppressive cells^[164]^. In particular, high levels of kynurein have been shown to predict resistance to PD-1 blockade and OS upon administration of nivolumab in advanced melanoma and renal cell carcinoma patients. Remarkably, in preclinical models IDO inhibitors had negligible effect when used alone but resulted synergic with anti-CTL-A4 and anti-PD-1 therapies in controlling cancer burden and favoring mice survival^[165]^. Therefore, different inhibitors are currently being studied in several tumor types as co-administered with anti-PD-1 antibodies (i.e., nivolumab or pembrolizumab) or anti-PD-L1 antibodies (i.e., atezolizumab or durvalumab)^[166]^ and the same approach is to be envisaged for ICIs-resistant CRCs.

An immunosuppressive role has been reported for VEGF, which can act via different mechanisms such as by decreasing the number of TILs and activating immune checkpoint molecules^[167]^. Notably, in a CRC mouse model it has been reported that addition of anti-angiogenic therapy reduced intrinsic resistance to an anti-PD-1 antibody via normalizing tumor vascularization and reducing hypoxia^[168]^. Several trials are ongoing to evaluate the efficacy of combining ICIs with an anti-angiogenic strategy plus chemotherapy, being the molecules tested bevacizumab and regorafenib.

Recently, several studies have shown that the gut microbiota plays a key role in regulating the efficacy of ICIs for cancer treatment of different solid tumors [Table 3, Figure 4]^[169]^. For example, the treatment with an anti-CTLA-4 monoclonal antibody controlled the growth of different tumor models (including a CRC model) in mice maintained in specific pathogen-free conditions and in the presence of specific *Bacteroides*, but not in germ-free mice and mice previously treated with antibiotics. Therefore, it appears that the gut microbiota might regulate the efficacy of immunotherapy with anti-CTLA-4. Accordingly, the introduction of two specific *Bacteroides* species (i.e., *B. thetaiotaomicron* and *B. fragilis*) in germ-free mice and antibiotics-treated mice restored the response to anti-CTLA-4 treatment^[170]^. Notably, a series of studies in melanoma mouse models and patients uncovered other microbiota components as instrumental in determining the response to ICIs. For example, in a melanoma mouse model the addition of the commensal gut *Bifidobacterium *to PD-L1 checkpoint blockade nearly abolished the growth of scarcely sensitive tumors^[171]^. Comparative analysis of the oral and gut microbiome of melanoma patients undergoing anti-PD-1 immunotherapy revealed significant differences in the diversity and composition of the microbiome of responders *vs*. non-responders. Similarly, the analysis of patient fecal microbiome samples showed significant diversity and relative abundance of bacteria of the Ruminococcaceae family in responding patients^[172]^. Analysis of baseline stool samples from metastatic melanoma patients before immunotherapy uncovered significant association between commensal microbial composition and clinical response. Bacterial species more abundant in responders included *Bifidobacterium longum*, *Collinsella aerofaciens*, and *Enterococcus faecium*. Accordingly, in a mouse model fecal microbiota transplantation from responding patients in germ-free mice improved tumor control, augmented T cell responses, and increased efficacy of anti-PD-L1 therapy^[173]^. Routy *et al*.^[174]^ showed that fecal microbiota transplantation from cancer patients who responded to ICIs into germ-free or antibiotic-treated mice improved the efficacy of anti-PD-1 therapy, whereas fecal microbiota transplantation from non-responding patients was ineffective. Profiling patients’ stool samples revealed low levels of the bacterium *Akkermansia muciniphila* in non-responding patients with lung and kidney cancer. Accordingly, oral supplementation of the bacteria after fecal microbiota transplantation with non-responders feces in antibiotic-treated mice restored the response to PD-1 blockade^[174]^. Regulation of gut microbiota composition or addition of the “right” bacteria to therapy seems therefore a very promising approach to overcome resistance, or at least significantly improve the response to ICIs.

## CONCLUSION

Intrinsic and acquired resistance to chemo-, targeted, and immune-therapy remain the core problem(s) to tackle for ameliorating the outcome of CRC patients, especially in the metastatic setting. Significant improvements and longer expectancy of life have been reached using a combination/integration of the different types of therapy, but only the identification of additional targetable liabilities to be exploited in specific settings will allow to concomitantly attack the tumor from different angles, hopefully leading to its eradication. In this sense, promising approaches are: the development of novel nanoparticles able to vehicle chemotherapy directly inside the cells avoiding the intake/efflux systems, often deregulated in resistant cells; the induction of synthetic lethality via the use of inhibitors of specific DNA damage checkpoints or DNA repair enzymes in tumors presenting defects in the DNA repair mechanisms; the use of BH-3 mimetics in tumors highly expressing anti-apoptotic members of the BCL2 family; the targeting of several kinases shown to sustain resistance to classic chemotherapy and also resistance to target therapy via activation of feed-back loops; the targeting of pathways crucial for cancer stem cells maintenance and viability; the targeting of several components of the TME, recognized to be responsible for an immune-privileged environment, among which the so-called immune checkpoints; and the modulation of the microbiota. For the most of these approaches an unavoidable task will be patient stratification according to specific biomarkers and/or genetic defects, indicating that precision medicine is the ultimate path to cover in order to identify effective therapies for each patient. In addition, given the high tumoral heterogenicity and the Darwinian pressure exerted by anti-cancer treatments, to prevent and/or overcome resistance it will be also essential to combine different types of therapies and using vertical approaches to target multiple nodes along the same pathway.

## DECLARATIONS

### Copyright

© The Author(s) 2022.
